# Milk Production and Climate Variability in a High-Andean Dairy System: A Longitudinal Case Study

**DOI:** 10.3390/ani16203169

**Published:** 2026-10-09

**Authors:** Guillermo Fausto Montenegro-Arellano, Luis Alfredo Carvajal-Pérez, Gladys Primavera Urgilés-Urgilés, Pablo David Quishpe-Sinailin, Anibal Eduardo Herrera-Lana

**Affiliations:** 1 Universidad Politécnica Estatal del Carchi, Antisana St. and Universitaria, Ave., Tulcán 040102, Carchi, Ecuador; guillermo.montenegro@upec.edu.ec (G.F.M.-A.);; 2 Instituto de Investigaciones Económicas, Universidad Central del Ecuador, Quito 170129, Pichincha, Ecuador

**Keywords:** autocorrelation, climate variability, dairy cattle, high-Andean system, milk production, single-case study, temperature, time series

## Abstract

Climatic variability can modify milk production through direct and indirect pathways, yet monthly farm time series also exhibit temporal dependence. A longitudinal, observational, single-case study was conducted in the San Francisco dairy system, Carchi, Ecuador, from March 2019 to December 2024. Of 70 calendar months, 64 had complete analytical information, and 58 were eligible for two-lag models. Ten model specifications were compared based on model fit, information criteria, residual diagnostics, seasonality assessment, and temporal validation. Mean daily milk production was 233.49 ± 47.90 L day^−1^. The selected model was a second-order autoregressive model with a calendar trend and mean temperature at *t* − 2 (adjusted R2 = 0.827; Akaike information criterion = 519.52; Bayesian information criterion = 529.83; root mean squared error = 19.56 L day^−1^). The temperature coefficient was 10.844 L day^−1^ °C^−1^ (*p* < 0.001 with robust standard errors). Annual harmonic terms did not improve model fit, and precipitation at *t* − 1 yielded only marginal improvement. The number of cows in production varied across 54 months (26.02 ± 4.11; range: 18–37). The observed temperature association should be interpreted at the system level; it does not demonstrate direct causality or individual cow productivity and requires external validation.

## Simple Summary

Mountain pasture-based dairy systems often exhibit substantial month-to-month fluctuations in milk production; however, these variations are not solely determined by climatic factors. This study analyzed monthly records from a dairy farm in Carchi, Ecuador, spanning March 2019 to December 2024 to determine whether weather conditions provide predictive value beyond the historical production trajectory. Mean temperature recorded two months prior was positively associated with mean daily herd milk output, whereas precipitation did not consistently improve model fit. Given the cool environment of the study site, this association was interpreted not as a heat-stress response, but rather as a signal compatible with delayed changes in pasture growth and utilization. The number of cows in production varied across 54 months (26.02 ± 4.11; range: 18–37). Therefore, the findings describe system-level dynamics rather than causal effects on individual cow productivity, requiring validation across additional farms.

## 1. Introduction

Dairy systems respond to climatic variability through direct effects on animals and indirect pathways related to forage growth, water availability, health constraints, and management decisions. Consequently, weather and climate conditions can alter animal performance, production stability, and the overall sustainability of dairy enterprises [1,2]. In dairy cattle, thermal conditions influence feed intake, metabolic responses, and overall productivity; however, the direction and magnitude of these associations depend on the production system and the environmental range analyzed [3,4,5,6].

The temperature–humidity index (THI) is commonly used to summarize thermo–hygrometric conditions [7,8]. However, in cool, high–altitude pasture–based systems, responses to climatic drivers may be mediated primarily through forage processes. Temperature influences thermal–time accumulation, regrowth, and pasture phenological development, whereas precipitation determines water input into the system-though its effect depends on rainfall event distribution, infiltration, drainage, and effective soil–water availability. These pathways can alter forage availability and quality, dry matter intake, and, ultimately, milk production.

Monthly production records also exhibit serial dependence. Production in any given month reflects its preceding trajectory, changes in herd structure, and feeding or management decisions that are rarely recorded at the same temporal resolution. Models that omit this autoregressive structure may erroneously attribute variations corresponding to internal system dynamics to climatic covariates. Time–series approaches have been applied to production forecasting in the Andes and to the characterization of seasonality in pasture–based systems [9]. Forecasting theory requires serial dependence to be explicitly represented and models to be validated using temporally ordered observations [10,11,12].

The objective of this study was to evaluate the association between climatic variability and mean daily milk production in the San Francisco high-Andean dairy system, Carchi, Ecuador, using a longitudinal single-case design. Additionally, we evaluated whether climatic covariates provided predictive value after controlling for production persistence, temporal trends, and potential annual seasonality. We hypothesized that a parsimonious autoregressive model incorporating a nonredundant climatic covariate would outperform a specification based solely on past production. The inferential scope was restricted to the analyzed system because monthly observations do not constitute independent replicates of farms or herds.

## 2. Materials and Methods

### 2.1. Study System and Data Sources

An observational, longitudinal, retrospective, single-case study was conducted using a monthly time series from the dairy system at the San Francisco Experimental Center of Universidad Politécnica Estatal del Carchi (UPEC), located in San Pedro de Huaca Canton, Carchi Province, Ecuador. The research center is located at 2930 m above sea level (0°37′ N, 77°43′ W), in a cool, humid mountain environment.

The herd was managed under a pasture-based system involving crossbred dairy cattle, rotational grazing, paddock-use planning based on forage recovery, pasture fertilization, and machine milking with routine production recording [13]. These operational features describe the general management of the production unit and should not be confused with monthly time-series covariates. Continuous records of biomass, forage quality, dry matter intake, supplementation, physiological status, or specific management events were not available across the entire study period.

Climatic data were obtained exclusively from the San Francisco Automatic Weather Station (EMA-San Francisco, by its Spanish acronym), located within the dairy production unit. The primary records consisted of sub-daily observations collected at variable sampling frequencies and subsequently aggregated to a daily scale. Air temperature, relative humidity, precipitation, solar radiation, wind speed, and atmospheric pressure were evaluated; the temperature-humidity index (THI) was calculated using the station’s temperature and relative humidity recordings. At the monthly scale, air temperature, relative humidity, wind speed, atmospheric pressure, and THI were averaged, whereas precipitation and solar radiation were aggregated as monthly cumulative totals.

Monthly milk production data were obtained from routine farm records. The study period comprised 70 calendar months (March 2019 to December 2024). Six months lacked a complete pair of production and climatic data: November and December 2020; January, February, and March 2021; and August 2021. The first five months constituted a consecutive gap, whereas August 2021 represented an isolated missing data point. Because the integrated datasets did not allow the missing values to be unequivocally attributed to production records, climatic records, or both, these months were classified as lacking full analytical overlap. No imputation, interpolation, or zero-substitution techniques were applied.

### 2.2. Response Variable and Temporal Traceability

Monthly milk production was converted into mean daily production to account for variations in calendar month duration:

(1) $$y_{t}=\frac{Y_{t}}{d_{t}}$$

In Equation (1), *y_t_* represents the mean daily milk production estimated for month *t* (L day^−1^), *Y_t_* is the total monthly milk production (L), and *d_t_* is the number of calendar days in month *t*. This transformation allows for direct comparison across months on a standardized temporal scale.

Lagged variables were constructed under strict calendar continuity (Table 1). A value at *t* − 1 was considered eligible only when a valid observation existed for the immediately preceding month; similarly, for *t* − 2, both preceding months were required to be consecutively present. The 64 valid months formed three continuous temporal segments: March 2019–October 2020, April–July 2021, and September 2021–December 2024. Consequently, the effective sample size was reduced to 61 observations for *t* − 1 model and 58 observations for *t* − 2 model; this reduction did not reflect additional missing data, but rather the structural impossibility of constructing lagged terms at the beginning of each temporal segment.

The number of lactating cows was available for 54 months and served as a complementary proxy for the herd’s productive scale. Due to its incomplete temporal coverage, this variable was excluded from the main model comparison (N = 58). Instead, its variability was characterized by using descriptive statistics and considered as contextual information when interpreting milk aggregate production.

### 2.3. Data Exploration and Lag Definition

Descriptive statistics were computed for all quantitative variables. Pearson and Spearman correlation coefficients were evaluated for exploratory purposes, as conventional significance tests assume independence among observations [14]. Data auditing—including verification of ranges, units, and missing values—followed a structured data–exploration protocol [15]. Collinearity was assessed using correlation matrices and variance inflation factors (VIF), which were interpreted with respect to model purpose and coefficient stability [16].

The lag window was defined *a priori* as zero to two months prior to model selection. The contemporaneous lag (*t*) evaluated immediate associations, whereas *t* − 1 and *t* − 2 represented time scales consistent with indirect pathways mediated by pasture growth, recovery, and utilization. Because the production system maintained an approximate 36-day grazing rotation interval, a maximum two-month window encompassed one to two complete cycles of forage recovery and utilization. This rationale aligns with the known influence of thermal time on the growth and nutritive value of temperate grasses and reinforces the critical role of rest periods in high–Andean pastures [17,18].

The specific selection of *t* − 2 was not *a priori* hypothesis. Lags *t*, *t* − 1, and *t* − 2 were compared within the predefined window, and the two–month lag was retained during empirical selection because it provided the best balance among model fit, information criteria, parsimony, parameter stability, residual diagnostics, and temporal performance. Accordingly, *t* − 2 is interpreted as a candidate time scale supported within the set of evaluated alternatives rather than as an exact physiological latency of 60 days. The *t* − 1 specification did not provide the same joint support across model fit, information criteria, residual diagnostics, and temporal validation as *t* − 2. Because adjacent monthly climatic variables may be correlated, this comparison is interpreted as empirical model–selection evidence rather than proof of a discrete two–month biological delay.

Evaluating multiple covariates and lags within a short time series increases the risk of spuriously significant associations. To mitigate this risk, an unrestricted lag search was avoided, redundant predictor combinations were excluded, finalist models were compared using a common sample, and selection did not rely solely on *p*–values, but rather on the convergence among biological, statistical, and predictive criteria.

### 2.4. Variable Selection and Candidate Models

Variable selection was performed in two stages. During the exploratory phase, mean temperature, relative humidity, precipitation, solar radiation, wind speed, and atmospheric pressure recorded at the EMA–San Francisco station were evaluated, and THI was derived from temperature and relative humidity. Not all candidate variables were retained for formal model comparison.

Mean temperature and THI were evaluated as alternative thermal descriptors and were not included simultaneously due to collinearity. Relative humidity was used to compute THI and was excluded from models that already incorporated thermal indicators. Wind speed and atmospheric pressure were restricted to the exploratory stage because their inclusion failed to yield meaningful improvements in model fit, parsimony, or residual diagnostics. Conversely, precipitation and solar radiation were initially retained given their biological plausibility as indirect drivers of water availability and forage biomass growth.

Ten structurally distinct specifications were formally evaluated (Table 2). These included baseline reference models, specifications incorporating climatic subsets at lags *t*, *t* − 1, and *t* − 2, alternative thermal formulations (mean temperature vs. THI), extensions with precipitation, and models explicitly incorporating annual seasonality. Predictors were excluded from subsequent modeling stages if they exhibited high redundancy, failed to improve Akaike information criterion (AIC), Bayesian information criterion (BIC), or root mean squared error (RMSE), degraded residual diagnostics, induced parameter instability, or increased model complexity without enhancing predictive performance. Individual statistical significance was treated as a complementary criterion rather than an absolute inclusion threshold. First-order [AR(1)] and second-order [AR(2)] autoregressive models were compared as alternative dynamic structures, with AIC, BIC, and RMSE serving as primary model-selection criteria.

The general model structure for comparison was specified as follows:

(2) $$y_{t}\;=\;\beta_{0}\;+\;\varphi_{1}\;y_{t-1}\;+\;\beta_{1}\;t\;+\;\beta_{2}\;x_{t-k}\;+\;\varepsilon_{t}$$

In Equation (2), x represents the vector of non-redundant climatic covariates, $\beta_{2}$ is the corresponding vector of regression coefficients, and k∈{0,1,2} denotes the lag order. When residual autocorrelation persisted, the model was extended by incorporating a second autoregressive term.

The final selected model was specified as:

(3) $$y_{t}\;=\;\beta_{0}\;+\;\varphi_{1}\;y_{t-1}\;+\;\varphi_{2}\;y_{t-2}+\beta_{1}t\;+\;\beta_{2}\;z_{t}\;+\;\varepsilon_{t}$$

In Equation (3), *t* represents the deterministic linear trend and *z_t_ = T_t_*_−2_ denotes the mean temperature recorded two months prior. Coefficient inference was based on heteroskedasticity– and autocorrelation–consistent (HAC) standard errors specified with three lags [19,20,21]. Model selection jointly considered adjusted R^2^, AIC, BIC, RMSE, biological plausibility, collinearity, parsimony, parameter stability, residual diagnostics, and temporal validation [22,23,24].

### 2.5. Temporal Structure, Diagnostics, Sensitivity, and Validation

The temporal structure was evaluated through three main components: autoregressive persistence, a linear trend, and potential annual seasonality. Serial dependence was examined using the autocorrelation function (ACF) and by comparing AR(1) and AR(2) structures. The trend was modeled using a linear time index intended to control gradual long-term changes not captured by the observed covariates.

Potential residual annual seasonality was evaluated using Fourier harmonic terms with a 12-month period:

(4) $$S_{t}=\gamma_{1}sin(\omega t)+\gamma_{2}cos(\omega t)$$

In Equation (4), *S_t_* represents the seasonal component added to the M–T specification, and ω = 2π/12 corresponds to the annual angular frequency. This harmonic formulation required only two parameters rather than eleven monthly dummy variables, thereby mitigating the risk of overfitting given the effective sample size of 58 observations (N = 58). Seasonality was evaluated based on the individual and joint significance of the sine and cosine terms, changes in AIC and BIC, and the Ljung–Box test applied to residuals at lag 12.

Model diagnostics included Durbin–Watson statistic [25], Ljung–Box tests at lags 1, 6, and 12 [26], the augmented Dickey–Fuller (ADF) test [27], Breusch–Pagan test [28], ARCH-LM test [29], and Jarque–Bera test [30], as well as Cook’s distance for influential observations. Potential structural instability was explored through chronological inspection and leave–one–out cross–re-estimation. Multiple structural breakpoints and non-linear trends were not estimated due to the limited series length and the risk of overfitting; therefore, the linear trend was interpreted as a parsimonious control for gradual change rather than as evidence of strict temporal linearity.

Temporal validation was performed by fitting candidate models to historical records available through December 2023 and evaluating 12 conditional out-of-sample forecasts, corresponding to each month of 2024. The training sample comprised 46 observations, and the validation sample comprised 12 observations. Predictive accuracy was evaluated using root mean squared error (RMSE), mean absolute error (MAE), and mean absolute percentage error (MAPE), which were compared against a naive benchmark model based on observed production at *t* − 1 [31,32,33].

### 2.6. Inferential Scope and Unobserved Variables

This study focused on a single diary production unit. Although the 58 monthly observations in the primary model permitted robust estimation of within-system associations, they did not account for between-farm variability. Consequently, estimated temperature coefficients reflect system-specific parameters for the San Francisco dairy herd over the 2019–2024 period rather than population-level effects generalizable across all high–Andean dairy systems [34,35,36].

Furthermore, the primary database lacked continuous monthly records for pasture biomass, forage nutritional quality, dry matter intake, individual cow milk yield, days in milk, parity, health status, strategic supplementation, or management interventions. Additionally, the number of lactating cows was available for only 54 months. These constraints preclude complete isolation of herd–scale operational effects from underlying pasture or animal-level physiological mechanisms; therefore, estimated parameters are interpreted as conditional associations rather than unconfounded causal relationships [34].

All statistical analyses and time–series modeling were implemented in *Python* (version 3.13.5) using *pandas* (v2.2.3), *NumPy* (v2.3.5), *SciPy* (v1.17.0), and *statsmodels* (v0.14.6). Dynamic linear models were estimated via ordinary least squares (OLS), with coefficient inference based on Newey–West heteroskedasticity– and autocorrelation–consistent (HAC) standard errors with a bandwidth of three lags. Model selection integrated adjusted *R*^2^, AIC, BIC, and RMSE criteria. Residual diagnostics comprised Durbin–Watson, Ljung–Box, augmented Dickey–Fuller (ADF), Breusch–Pagan, ARCH-LM, and Jarque–Bera tests, alongside Cook’s distance for influential observation screening. Out-of-sample predictive performance was evaluated using 12-month conditional temporal validation for 2024.

### 2.7. Use of Generative AI Tools

OpenAI ChatGPT (GPT-5.6 Sol) was used during manuscript preparation exclusively for language editing, sentence restructuring, redundancy reduction, and grammatical refinement. OpenAI ChatGPT Images 2.5 was used to assist in the visual design and structural layout of the graphical abstract. These artificial intelligence tools were not used to generate, simulate, or alter empirical data, perform statistical analyses, or formulate scientific interpretations and conclusions. All scientific content, analytical logic, and final wording were thoroughly reviewed, verified, and approved by the authors, who take full responsibility for the integrity of the manuscript.

## 3. Results

### 3.1. Temporal Data Availability and Descriptive Behavior

**Figure 1:**
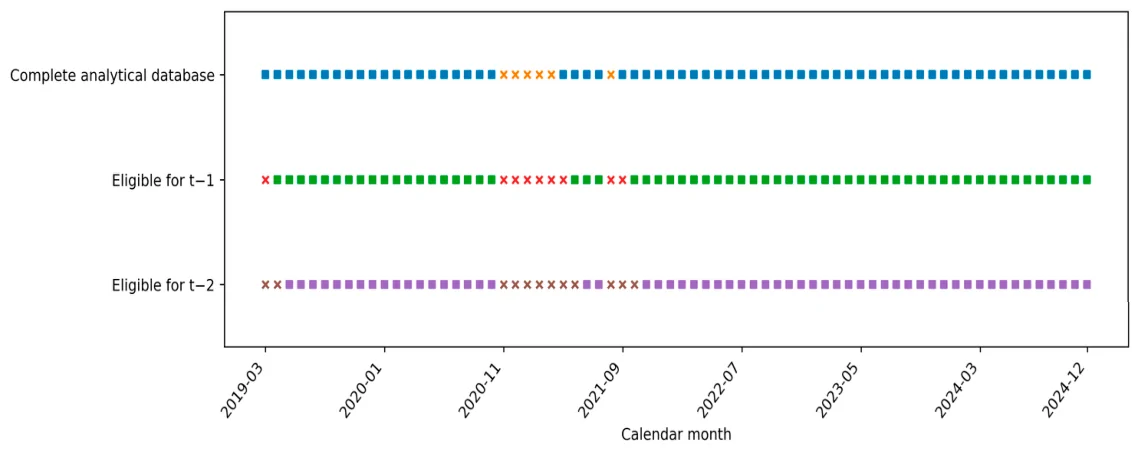
Monthly data availability and eligibility for temporal lag construction. Note. Squares represent available or eligible months; crosses indicate missing or non-eligible months resulting from calendar discontinuities. Primary analytical database: n = 64; *t* − 1 lagged sample: n = 61; *t* − 2 effective sample: n = 58.

The observation window comprised 70 calendar months, of which 64 had complete analytical information. The six non-eligible months were concentrated in two distinct gaps: a continuous five-month interruption from November 2020 through March 2021 and an isolated missing record in August 2021. Calendar–based lag construction reduced the effective sample to 61 observations for *t* − 1 and 58 for *t* − 2. This balanced 58-observation sample (N = 58) served as the common baseline for evaluating models with up to two dynamic lags, thereby preventing discrepancies in information criteria attributable solely to sample size variations (Figure 1).

Table 3 describes: Mean daily milk production averaged 233.49 L day^−1^ (SD = 47.90; CV = 20.52%), with monthly values ranging between 158.77 and 396.53 L day^−1^. Precipitation exhibited the highest climatic variability (CV = 78.84%), ranging from 0.00 to 287.10 mm month^−1^. Mean monthly temperature ranged from 10.74 to 13.52 °C, whereas THI ranged between 51.78 and 56.40.

### 3.2. Serial Dependence, Collinearity, and Model Comparison

Mean daily milk production exhibited strong persistence, with a first-order autocorrelation of 0.851. The Ljung–Box test rejected the white-noise null hypothesis up to lag 12 (Q(12) = 113.79; *p* < 0.001), whereas the ADF test rejected the unit–root null hypothesis at the 5% significance level (ADF statistic = −3.214; *p* = 0.019). Collectively, these diagnostics confirmed a stationary series featuring serial dependence, justifying a dynamic specification.

In exploratory bivariate analyses, contemporaneous precipitation displayed negligible correlation with milk yield (*r* = 0.026; nominal *p* = 0.838). Solar radiation showed an inverse correlation (*r* = −0.412; nominal *p* < 0.001), whereas atmospheric pressure was positively correlated (*r* = 0.497; nominal *p* < 0.001); these nominal *p*-values were interpreted descriptively due to serial autocorrelation. Mean temperature and THI exhibited severe collinearity (*r* = 0.997), yielding VIF values exceeding 3000 when fitted jointly with relative humidity. In the parsimonious specification, the VIF for mean temperature was 2.08, and the maximum VIF across predictors in the final model was 4.72.

Within the predefined 0–to-2–month window, contemporaneous and one–month–lag specifications lacked sufficient joint performance to be retained. The two-month lag (*t* − 2) received stronger empirical support during model comparison and was integrated into the candidate thermal models; thus, its selection was data-driven through model evaluation criteria rather than imposed *a priori*.

**Figure 2:**
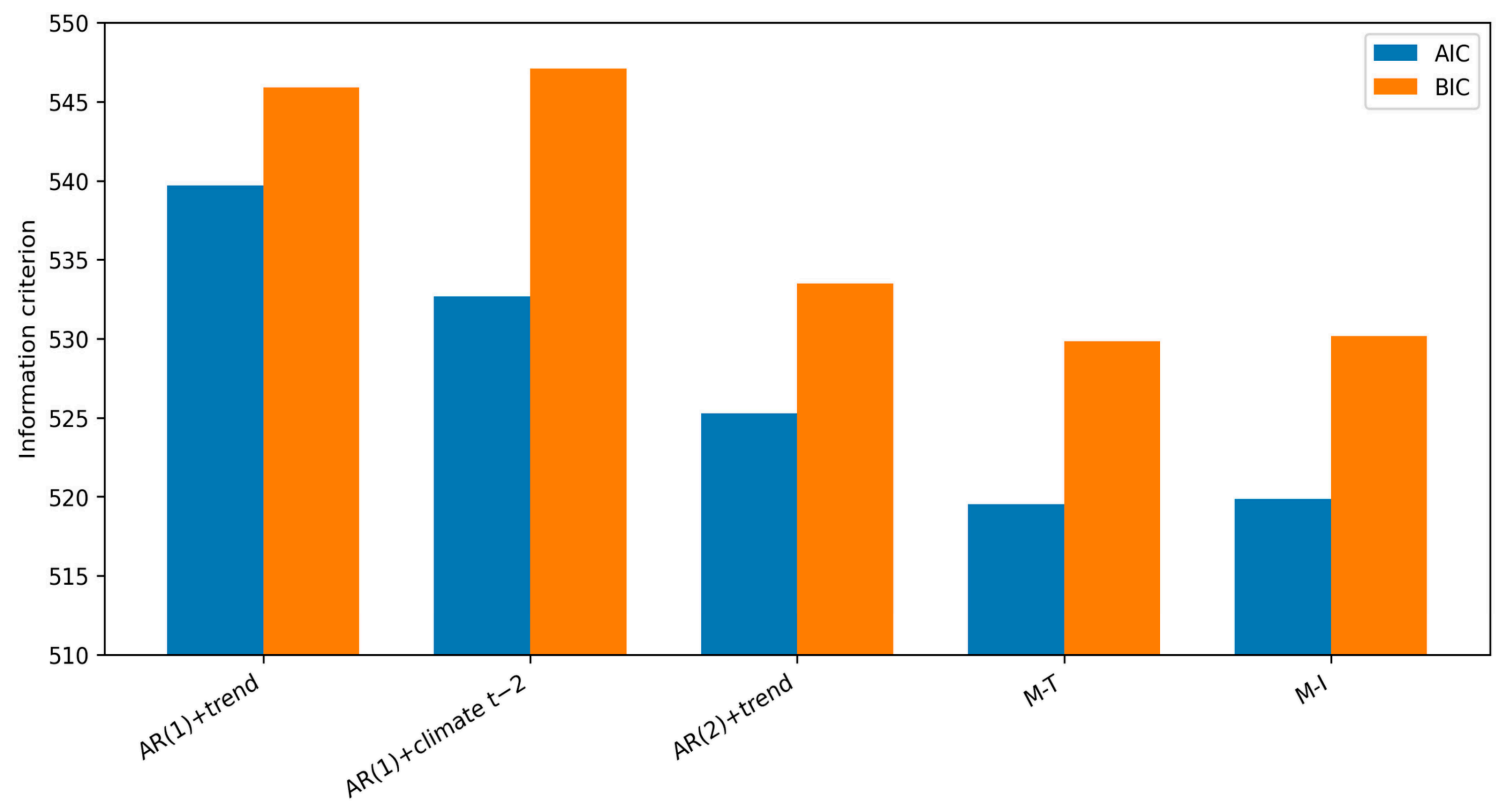
Comparison of AIC and BIC values across candidate finalist dynamic models. Note. Lower criterion values indicate superior relative balance between model fit and statistical complexity within the candidate model set.

Of the 10 candidate model specifications, five were retained as finalists for formal numerical comparison. Among these, the M–T model specification achieved the lowest AIC, BIC, and RMSE values within the common balanced sample (N = 58) (Table 4; Figure 2).

### 3.3. Final Model and Residual Diagnostics

**Figure 3:**
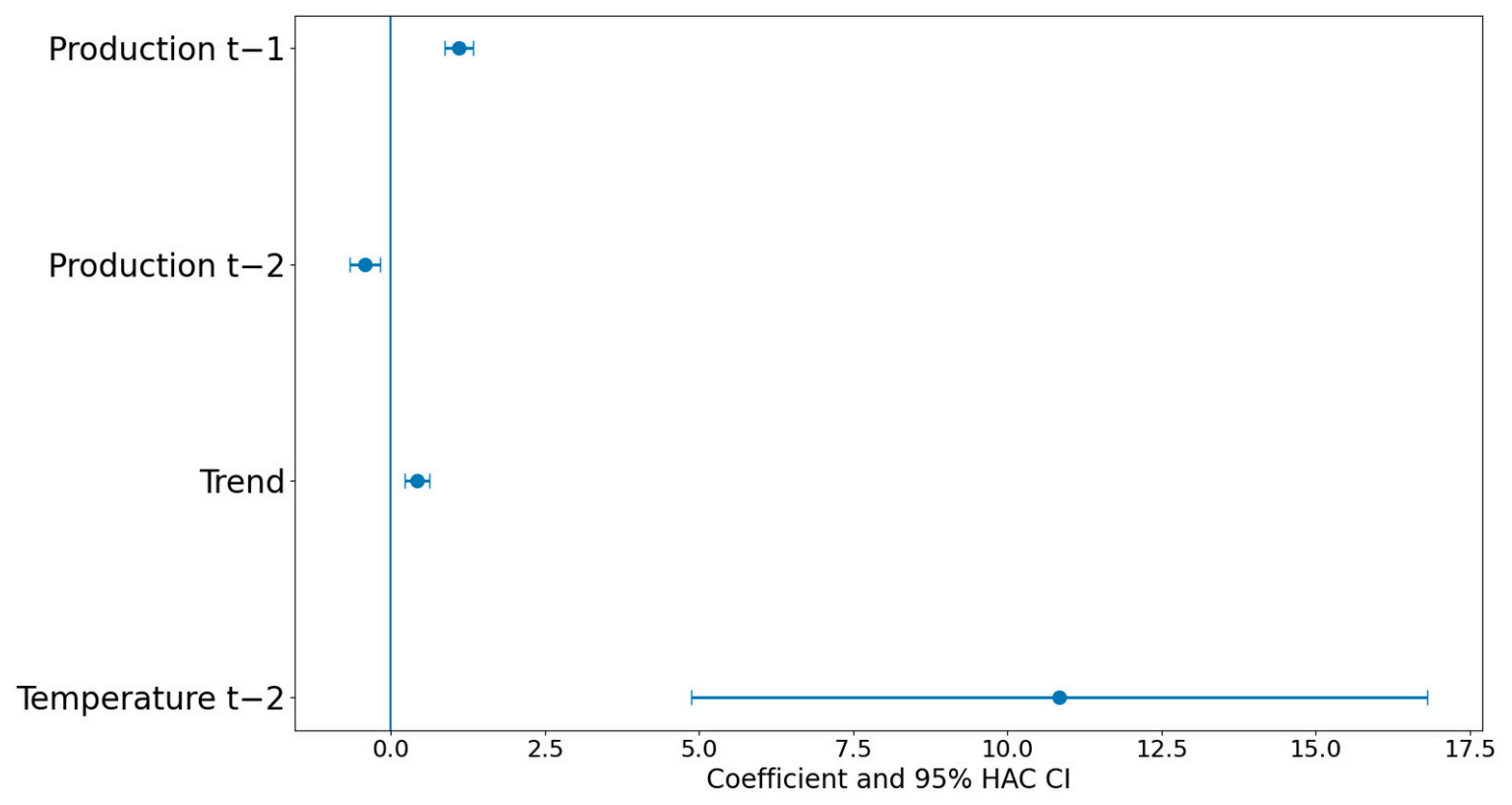
M–T model coefficients and 95% HAC confidence intervals. Note. All coefficients are reported in their original measurement units; consequently, point estimate magnitudes are not directly comparable across predictors.

The selected M–T model achieved an adjusted *R*^2^ of 0.827 and an in-sample RMSE of 19.56 L day^–1^. Mean temperature at lag 2 (*t* − 2) yielded an estimated coefficient of 10.844 L day^–1^ °C^−1^ (HAC *p* < 0.001). The roots of the characteristic polynomial associated with the autoregressive process lie strictly within the unit circle (eigenvalue moduli of 0.640), confirming dynamic stability. Table 5 summarizes the parameter estimates, HAC standard errors, and residual diagnostic tests; Figure 3 displays the coefficient estimates alongside their 95% HAC confidence intervals.

### 3.4. Variation in the Number of Cows in Production

The number of lactating cows did not remain constant during the study period. Across 54 months with available records, herd size averaged 26.02 ± 4.11 cows (range: 18–37 cows; CV = 15.81%).

Because herd size data did not cover the entire timeframe evaluated in the primary model comparison, herd size was omitted as an explanatory covariate in the final 58-observation dynamic model. Instead, it served as contextual information when interpreting aggregate system-level production. Consequently, the estimated temperature coefficient reflects total farm-level production response rather than individual cow productivity adjustments.

### 3.5. Seasonality, Precipitation, and Influence

Annual harmonic terms did not yield statistically significant explanatory power. The estimated sine term coefficient was −3.52 (HAC *p* = 0.464) and the cosine term was −2.69 (HAC *p* = 0.371), with a joint test yielding F(2,51) = 0.51 (*p* = 0.606). The extended seasonal model yielded higher information criteria (AIC = 522.39; BIC = 536.81) compared to the benchmark M–T specification (AIC = 519.52; BIC = 529.83). Furthermore, the Ljung–Box test at lag 12 revealed no significant annual residual autocorrelation (*p* = 0.542). Therefore, an explicit seasonal harmonic structure was not justified after accounting for autoregressive persistence, temporal trend, and lagged temperature.

Including precipitation at lag 1 (*t* − 1) slightly decreased AIC from 519.52 to 518.61 and reduced validation RMSE from 31.00 to 30.05 L day^–1^. However, BIC increased from 529.83 to 530.97, and the partial F–test was not statistically significant (*p* = 0.108). Similarly, precipitation at lag 2 (*t* − 2) failed to simultaneously improve information criteria, diagnostic tests, and predictive performance. Consequently, precipitation at *t* − 1 was retained strictly as a sensitivity analysis rather than incorporated into the primary model.

Four observations exceeded the heuristic Cook’s–distance threshold of 4/*n*: October 2023, along with September, November, and December 2024. Leave–one–out sensitivity re-estimations consistently preserved the positive sign and statistical significance of the temperature coefficient, with point estimates ranging between 10.36 and 12.62 L day^–1^ °C^−1^ across all iterations. No influential points were discarded, as all empirical records were verified as plausible representations of system variability.

### 3.6. Temporal Validation

**Figure 4:**
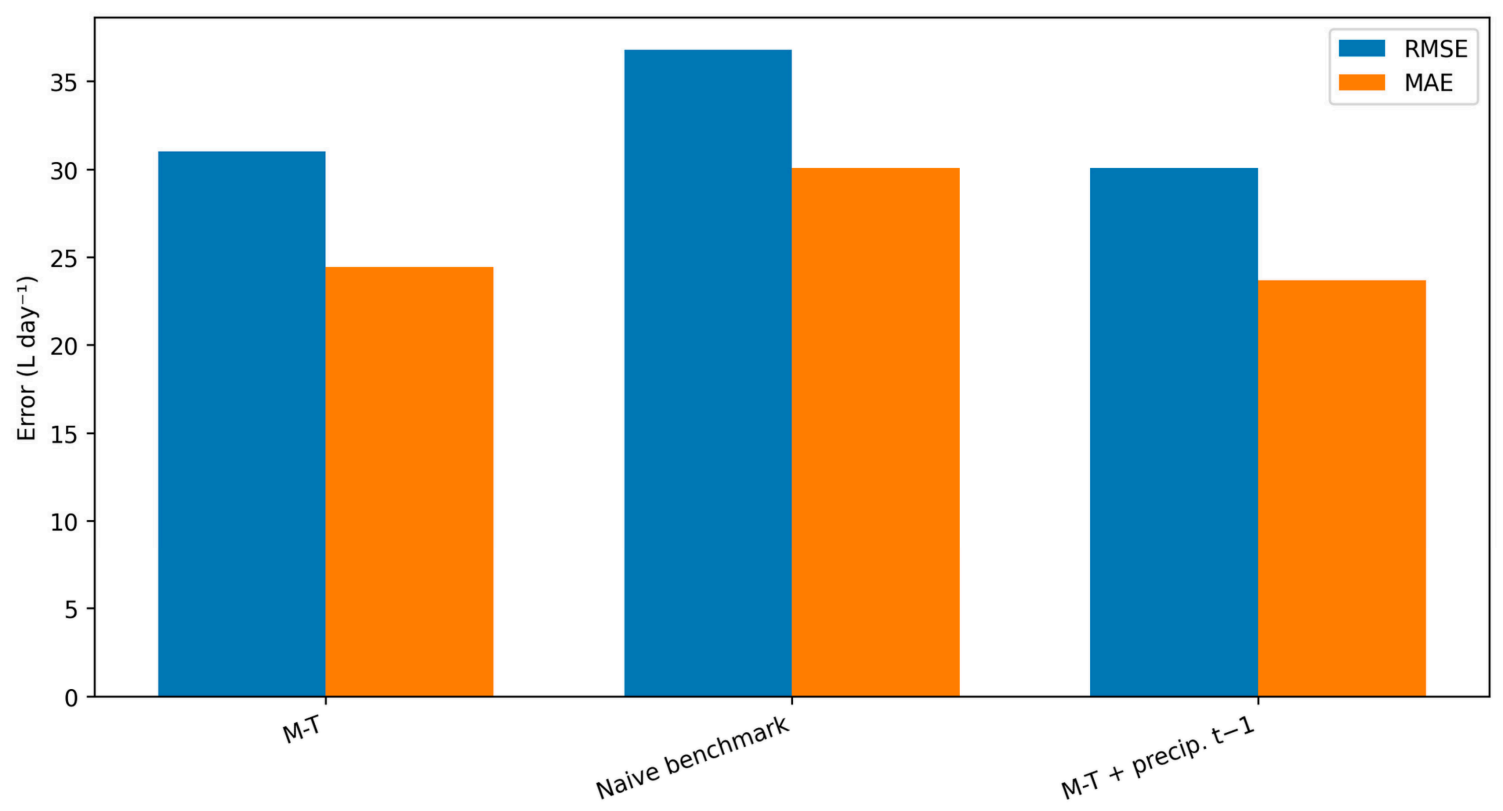
Performance of conditional temporal validation during 2024. Note. Both RMSE and MAE are expressed in L day^–1^. The naive persistence benchmark predicted the current monthly output using the observed production from the immediately preceding month.

In the conditional temporal validation for 2024, the M–T model achieved an RMSE of 31.00 L day^–1^, an MAE of 24.45 L day^–1^, and a MAPE of 8.18%. The naive persistence benchmark (based on production from the previous month) yielded RMSE = 36.79 L day^–1^, MAE = 30.06 L day^–1^, and MAPE = 10.33%. The specification incorporating precipitation at lag 1 (*t* − 1) achieved RMSE = 30.05 L day^–1^ and MAE = 23.68 L day^–1^, but failed to consistently outperform the M-T benchmark across the remaining model selection criteria (Figure 4).

## 4. Discussion

### 4.1. Temporal Structure, Trend, and Seasonality

The high persistence observed in milk production aligns with the biological continuity of lactation cycles and the relative month-to-month stability of herd management and feeding strategies. The AR(2) structure effectively captures this dynamic memory, thereby mitigating the risk of spuriously attributing intrinsic time-series dynamics to climatic covariates. Furthermore, the linear calendar trend captured unobserved gradual baseline shifts, although its estimated coefficient aggregates multiple unmeasured deterministic drivers.

The lack of statistical significance among annual harmonic terms does not imply the absence of seasonal seasonality in climate, pasture dynamics, or milk output. Instead, it indicates that once autoregressive persistence, temporal trend, and lagged temperature are accounted for, no sufficiently stable residual annual cycle remains to justify additional parameter complexity. The statistical agreement across the joint Fourier test, the elevated AIC and BIC values for the expanded specification, and the absence of residual autocorrelation at lag 12 together confirm that the observed temperature association is not merely an artifact of shared seasonal variation [37].

Nevertheless, the effective sample size of 58 observations constrains statistical power to disentangle complex cycle patterns, interannual variability, structural breaks, or non-linear long-term trends. Consequently, the specified linear trend should be interpreted as a parsimonious deterministic control rather than evidence of strictly linear systemic evolution over time. Longer time-series data would enable formal testing of structural change points and more flexible non-parametric temporal functions while minimizing the risk of overfitting.

### 4.2. Biological Interpretation of Lagged Mean Temperature

The positive association observed with mean temperature at lag 2 (*t* − 2) does not reflect classic heat stress dynamics. The observed thermal ranges (10.74–13.52 °C; THI = 51.78–56.40) describe a cool environment well below the critical thresholds associated with heat–stress-induced production losses in dairy cattle [3,4,5,6,7,8]. In temperate and high-altitude pastoral systems, weather impacts on milk yield are strongly mediated by herd management and grazing access [38].

A plausible mechanism compatible with this production system involves an indirect pathway mediated by pasture dynamics. Ambient temperature regulates thermal–time accumulation, leaf appearance and elongation rates, sward regrowth, and phenological development in temperate grasses, modifying both forage biomass yield and nutritional quality [17]. In high–Andean dairy systems, pasture rest intervals directly influence carrying capacity and forage utilization efficiency [18], which in turn dictate dry matter intake, energy balance, and milk production in grazing cows [39,40].

The proposed conceptual sequence follows: climate → forage growth and maturity → diet availability and nutritional quality → dry matter intake → milk production. Given the approximate 36-day rotation cycle between grazing events, a thermal condition recorded in month *t* − 2 can plausibly influence pasture development during the subsequent growth cycle, with production responses materializing in the aggregate monthly output. Therefore, lag *t* − 2 does not represent a strict 60-day physiological latency, but rather an operational temporal scale reflecting one to two pasture recovery cycles combined with monthly data aggregation.

This mechanism interpretation remains a working hypothesis. Forage biomass, nutritional composition, and dry matter intake were not measured monthly; consequently, forage mediation cannot be empirically proven with the current dataset. In addition, the specific *t* − 2 lag was selected empirically within a predefined biological window, and the evaluation of multiple variables and lags may favor sample–specific associations. Search restrictions, information criteria, diagnostics, and temporal validation reduce, but do not eliminate, this uncertainty. Lag 2 (*t* − 2) should be regarded as a testable temporal hypothesis for future studies.

### 4.3. Precipitation and Representation of the Hydrological Process

The absence of a robust predictive contribution from rainfall does not imply biological irrelevance. Although precipitation exhibited the highest climatic variability in the dataset (CV = 78.84%), monthly cumulative totals represent gross water input rather than net effective soil-water availability for plant growth. Pasture response depends heavily on rainfall intensity and daily distribution, infiltration, surface runoff, actual evapotranspiration, subsoil drainage, local topography, and physical soil properties.

Furthermore, the empirical relationship between rainfall and pasture growth is intrinsically non-linear. Severe water deficits restrict leaf expansion and regrowth, whereas excessive moisture or soil waterlogging reduces photosynthesis and root growth [41,42]. In pasture–based systems, soil saturation can also reduce effective access to paddocks, increase treading damage, and modify rotation or supplementation decisions [43]. In addition, temperature and precipitation may interact nonlinearly in grassland responses [44].

In this context, contemporaneous rainfall or short monthly lags of one or two months may fail to capture cumulative hydrological deficits or longer-term soil-water memory. The marginal improvement observed when introducing precipitation at lag 1 (*t* − 1) is compatible with this complexity but was not sufficiently consistent to justify inclusion in the primary model. Future studies should incorporate daily precipitation, evapotranspiration, water balance, direct measurements of soil moisture, drainage, trafficability, and treading damage. Future work could also evaluate the number of rain days per month, cumulative water balance, and soil saturation thresholds to better represent hydrological exposure in Andean pastures.

### 4.4. Herd Size, Animal Science, and Scope of the Case Study

Fluctuations in the number of lactating cows represent a critical determinant when interpreting aggregate farm-level milk output. Because monthly herd–size records were incomplete, herd size could not be incorporated consistently into the primary model comparison. Accordingly, the temperature coefficient from the aggregate model should not be interpreted as an effect on liters per cow per day. This limitation reinforces the need for individual–level records and herd–composition data in future studies. The available data do not establish whether the *t* − 2 temperature association persists for herd–average milk yield per cow, because cow–count records were available for only 54 months and were not included in the primary lagged analytical matrix.

From an animal–science perspective, the observed dynamics integrate animal, forage, and management processes. The specificity of mountain milk depends largely on the forage system [45] and seasonal changes in diet [46], whereas studies conducted at different elevations show physiological and nutritional conditions in mountain systems [47]. Therefore, the value of the model lies in representing the internal dynamics of a specific pasture-based unit rather than in isolating a single causal mechanism.

The study included a single production unit. Monthly observations represent temporal replication within San Francisco but not independent replicates across farms or herds [48,49]. Accordingly, the estimated coefficient is specific to the system and period studied. External validity requires replication of the analysis on farms with different microclimates, herd sizes, forage bases, and management practices [50], ideally using hierarchical or panel models that separate temporal and between-farm variation.

### 4.5. Practical Application and Research Priorities

The empirical specification can be updated monthly to benchmark observed milk output against expected trajectories and identify deviations requiring management review. Operational use should recognize that predictions are conditional on production observed in the two preceding months and therefore do not represent fully ex ante scenarios. Weather information may serve as an early indicator for planning forage budgets, grazing rotations, and supplementation, but this utility requires external validation.

Future research should continuously record biomass, canopy height and leaf stage, botanical composition, protein, fiber, metabolizable energy, dry matter intake, supplementation, number of cows milked, individual production, days in milk, body condition, health, and reproduction. Such data would allow explicit testing of the climate–forage–diet–intake–milk production sequence and the development of bioeconomic models with greater explanatory capacity and decision–making utility.

## 5. Conclusions

Mean daily milk production in the San Francisco system exhibited marked temporal persistence. The AR(2) specification incorporated with a deterministic calendar trend and two-month lagged mean temperature achieved the optimal balance among goodness-of-fit, parsimony, structural stability, residual diagnostics, and conditional temporal validation. No residual annual seasonality was detected, and precipitation failed to provide consistent predictive improvement under the linear forms and lag structures evaluated. The positive temperature association is restricted to the observed microclimatic range and aligns with delayed mechanisms of pasture growth and forage utilization, though it does not establish unconfounded causality. Although lactating herd size fluctuated during the monitoring period, incomplete records precluded its baseline integration; thus, the temperature parameter reflects aggregate system yield rather than individual cow productivity. Given the single-case design, generalization requires longer series, cow-level longitudinal data, and cross-farm validation.

## Figures and Tables

**Table 1:** Traceability of temporal data availability and effective sample size.

Stage	Eligibility Criterion	*N*	Loss from Previous Stage	Cumulative Loss
Calendar window	March 2019–December 2024	70	—	—
Complete analytical database	Concurrent availability of production and climatic data	64	6	6
Models with *t* − 1	Preceding month available and consecutive	61	3	9
Models with *t* − 2	Two preceding months available and consecutive	58	3	12
Final model comparison	Balanced sample with two lags	58	0	12

Note. Months lacking analytical alignment were November 2020–March 2021 and August 2021. Additional sample reduction resulted from calendar–based lag construction. No missing observations were imputed.

**Table 2:** Traceability of the 10 candidate specifications.

ID	Structure	Climatic Variables	Lag	Decision
M0	AR(1) + trend	No climate	—	Baseline dynamic reference; excluded due to residual autocorrelation
M1	AR(1) + climatic subset	*T*, precipitation, radiation	*t*	Contemporaneous effects; not retained
M2	AR(1) + climatic subset	*T*, precipitation, radiation	*t* − 1	One–month delayed effects; not retained
M3	AR(1) + climatic subset	*T*, precipitation, radiation	*t* − 2	Two–month delayed effects; finalist
M4/M–T	AR(2) + trend + T	Mean temperature	*t* − 2	Primary thermal candidate; selected
M5/M–I	AR(2) + trend + THI	THI	*t* − 2	Thermal sensitivity candidate; finalist
M6	AR(2) + trend	No climate	—	AR(2) reference; finalist
M7	M–T + precipitation	Precipitation	*t* − 1	Hydrological sensitivity; partial improvement
M8	M–T + precipitation	Precipitation	*t* − 2	Hydrological sensitivity; no joint improvement
M9	M–T + sine + cosine	Harmonic components	annual (12-month)	Formal seasonality; not retained

Note. T, mean temperature. M–T denotes the model specification incorporating mean temperature, and M–I the alternative specification with THI. All meteorological variables were obtained from the EMA–San Francisco station.

**Table 3:** Descriptive statistics of the climate–production analytical database (*n* = 64).

Variable	Unit	n	Mean	SD	CV (%)	Minimum	Maximum
Mean daily milk production	L day^−1^	64	233.49	47.90	20.52	158.77	396.53
Precipitation	mm month^−1^	64	73.57	58.00	78.84	0.00	287.10
Mean temperature	°C	64	12.12	0.73	6.04	10.74	13.52
Relative humidity	%	64	90.31	3.50	3.88	77.79	94.56
Temperature–humidity index	Index	64	54.05	1.23	2.27	51.78	56.40

Note. SD, standard deviation; CV, coefficient of variation. Mean daily milk production was calculated by dividing total monthly production by the corresponding number of days in each month. Here, n = 64 represents the primary analytical dataset prior to lag truncation.

**Table 4:** Comparison of finalist dynamic model specifications (N = 58).

Model	*n*	Adjusted *R*^2^	AIC	BIC	RMSE (L day^−1^)	Ljung–Box *p* (12)
AR(1) + trend	58	0.747	539.71	545.89	24.09	0.004
AR(1) + climate (*t* − 2)	58	0.789	532.67	547.10	21.17	0.048
AR(2) + trend	58	0.806	525.27	533.51	20.91	0.822
M–T: AR(2) + trend + T *t* − 2	58	0.827	519.52	529.83	19.56	0.542
M–I: AR(2) + trend + THI *t* − 2	58	0.826	519.85	530.15	19.61	0.583

Note. All candidate models were estimated using the common balanced effective sample (N = 58). The M–T model was selected as the optimal specification based on the trade-off among goodness-of-fit, parsimony, parameter stability, residual diagnostic adequacy, and predictive performance.

**Table 5:** Coefficients and diagnostics of the M–T model.

Term or Diagnostic	Estimate	HAC SE/Statistic	*p*	Interpretation
Intercept	−76.282	44.223	0.090	Not significant at α = 0.05
Production (*t* − 1)	1.105	0.119	<0.001	Short-term production persistence
Production (*t* − 2)	−0.409	0.126	0.002	Second-order dynamic adjustment
Calendar trend	0.432	0.100	<0.001	Positive conditional temporal change
Mean temperature (*t* − 2)	10.844	3.044	<0.001	Positive thermal response within observed range
Durbin–Watson	2.156	—	—	No first-order residual autocorrelation
Ljung–Box (lag 12)	—	—	0.542	No residual autocorrelation up to 12 lags
Breusch–Pagan	—	—	0.111	Homoscedastic residuals
ADF test on residuals	−4.577	—	<0.001	Stationary residuals

Note. HAC standard errors were calculated using three lags. ADF, augmented Dickey–Fuller test.

## Data Availability

The analytical data supporting the results reported in this study are openly available in Figshare at https://doi.org/10.6084/m9.figshare.33806557.

## Abbreviations

The following abbreviations are used in this manuscript:

**Abbreviation**	**Definition**
ACF	Autocorrelation Function
ADF	Augmented Dickey–Fuller Test
AIC	Akaike Information Criterion
AR	Autoregressive
AR(1)	First-Order Autoregressive Model
AR(2)	Second-Order Autoregressive Model
ARCH	Autoregressive Conditional Heteroskedasticity
BIC	Bayesian Information Criterion
CI	Confidence Interval
CV	Coefficient of Variation
HAC	Heteroskedasticity– and Autocorrelation–Consistent
MAE	Mean Absolute Error
MAPE	Mean Absolute Percentage Error
M–I	Alternative Model Including the Temperature–Humidity Index
M–T	Selected Model Including Mean Temperature
OLS	Ordinary Least Squares
RMSE	Root Mean Square Error
SD	Standard Deviation
THI	Temperature–Humidity Index
UPEC	Universidad Politécnica Estatal del Carchi
VIF	Variance Inflation Factor
